# Construction of ZnTiO_3_/Bi_4_NbO_8_Cl heterojunction with enhanced photocatalytic performance

**DOI:** 10.1186/s11671-020-3292-4

**Published:** 2020-03-27

**Authors:** Zhaoqun Gao, Xiaofei Qu

**Affiliations:** College of Materials Science and Engineering, Qingdao University of Science and Technology, Zhengzhou Road 53, Qingdao, 266042 China

**Keywords:** ZnTiO_3_/Bi_4_NbO_8_Cl, Heterojunction, Photocatalysts, Photodegradation

## Abstract

Constructing heterojunction is an effective strategy to enhance photocatalytic performance of photocatalysts. Herein, we fabricated ZnTiO_3_/Bi_4_NbO_8_Cl heterojunction with improved performance via a typical mechanical mixing method. The rhodamine (RhB) degradation rate over heterojunction is higher than that of individual ZnTiO_3_ or Bi_4_NbO_8_Cl under Xenon-arc lamp irradiation. Combining ZnTiO_3_ with Bi_4_NbO_8_Cl can inhibit the recombination of photo-excited carriers. The improved quantum efficiency was demonstrated by transient-photocurrent responses (PC), electrochemical impedance spectroscopy (EIS), photoluminescence (PL) spectra, and time-resolved PL (TRPL) spectra. This research may be valuable for photocatalysts in the industrial application.

## Introduction

Photocatalysis has been attracting great interests in recent years, which already been applied in the fields of solar cells, water splitting, and water purification [1–4]. It has been reported that oxide based semiconductors are active photocatalysts [5], typified by TiO_2_ [6, 7], ZnO [8], and so on. However, individual pristine ZnO or TiO_2_ does not show gratifying photocatalytic performance. Specially, ZnTiO_3_ shows better performance in perovskite-type oxides. ZnTiO_3_ has been utilized in the fields of gas sensor and photocatalysis, etc. [9, 10]. However, the wide band gap of ZnTiO_3_ (3.1 ~ 3.65 eV) [9–13] limits its utilization of solar energy. On the other hand, the high recombination rate of photo-generated charges is another limitation factor. It is necessary to take measures to enhance its photocatalytic performance. One feasible and convenient route is that coupling ZnTiO_3_ with a type of narrow band gap semiconductor to form a heterojunction structure [14]. The narrow band gap semiconductor could behave as sensitizer to increase the light-harvesting ability and photocatalytic performance.

Bi_4_NbO_8_Cl, a promising candidate for increasing light-harvesting with several merits including narrow band gap (~ 2.38 eV), layered structure, appropriate potential of energy band [15–17], appears in the sight of researchers. Due to its low band gap energy and layered structure, this material could absorb light with a wavelength under 520 nm and benefit to charge transfer [18]. Some heterojunctions based on Bi_4_NbO_8_Cl have been prepared, such as Bi_2_S_3_/Bi_4_NbO_8_Cl [17] and g-C_3_N_4_/Bi_4_NbO_8_Cl [19]. Therefore, constructing ZnTiO_3_/Bi_4_NbO_8_Cl heterojunction may be a useful measure to enhance photocatalytic performance.

In this study, we fabricate a series of ZnTiO_3_/Bi_4_NbO_8_Cl heterojunction and evaluate the photocatalytic performance by RhB degradation under Xenon-arc lamp irradiation. Our results indicate that performance of the heterojunction is better than that of individual component. The formation of heterojunction could slow down the combination of electrons and holes, which leads to the enhanced degradation activity for RhB. The possible photocatalytic mechanism is discussed in details.

## Experimental

### Materials

Bismuth oxide (Bi_2_O_3_), ethanol (C_2_H_6_O), tetrabutyl titanate (C_16_H_36_O_4_Ti), acetic acid (CH_3_COOH), and zinc nitrate (Zn(NO_3_)_2_•6H_2_O) were obtained from Sinopharm Chemical Reagent Co., Ltd; bismuth oxychloride (BiOCl) and niobium pentoxide (Nb_2_O_5_) were obtained from Energy Chemical (Shanghai, China). All of the reagents used in this work are analytical grade and without further purification.

### Preparation of Bi_4_NbO_8_Cl

Bi_4_NbO_8_Cl was synthesized by ball mill mixing and solid state reaction methods. The mixing of materials was carried out in a planetary ball miller (Grinoer-BM4, China), equipped with corundum milling jar and corundum balls. Bi_2_O_3_ (18 g), BiOCl (12 g), and Nb_2_O_5_ (6 g) were weighted and mixed using ethanol (30 mL) as a dispersion solution in a milling jar, and fifty balls (10 mm diameters) were added and then ball milled for 2 h at 300 rpm. After grinding, the mixed reagents were dried at 60 °C for 12 h and calcined at 600 °C (heating rate of 5 °C/min) in air for 10 h. Finally, the yellow powders of Bi_4_NbO_8_Cl were obtained.

### Preparation of ZnTiO_3_

The sol-gel procedure was used to prepare ZnTiO_3_ powder. In a typical synthesis, 34 mL of tetrabutyl titanate (0.1 mol) was dissolved in 35 mL of ethanol to form a solution A. Five milliliters of deionized water, 15 mL of acetic acid (CH_3_COOH), and a certain amount of Zn(NO_3_)_2_•6H_2_O were successively dissolved in 35 mL of ethanol to form a solution B. Then, the solution B was added dropwise to the solution A under magnetic stirring. A transparent sol was obtained after the addition of stirring for 30 min which formed a gel over a rest period of 24 h. The gel was dried at 105 °C for 12 h, and then the resulting product was calcined at 600 °C for 3 h at the heating rate of 2 °C/min to obtain the final ZnTiO_3_ powders.

### Preparation of ZnTiO_3_/Bi_4_NbO_8_Cl heterojunction

In a typical experiment, 400 mg Bi_4_NbO_8_Cl and a certain amount of ZnTiO_3_ (mass ratio of ZnTiO_3_:Bi_4_NbO_8_Cl = 10%, 20%, 30%) were mixed and ground for 10 min, and then they were dispersed in 10 mL of ethanol, and followed by ultrasonic for 30 min. The resulting mixtures were dried at 60 °C for 12 h, and then calcined at 300 °C for 2 h. The as-fabricated samples were denoted as 10% BNZ, 20% BNZ, and 30% BNZ.

### Characterization

X-ray powder diffraction (XRD) measurements were recorded with a D-max 2500 XRD spectrometer (Rigaku), and the scan ranges were 10–80° with 10°/min. The morphologies of the as prepared samples were characterized by the scanning electron microscopy (SEM, JSM-6700F, JEOL, Japan) and transmission electron microscopy (TEM, JEM-2100, JEOL, Japan). The energy dispersive spectroscopy and elemental mapping analysis were obtained with the X-ray spectrometer equipped on the scanning electron microscope. UV-vis diffuse reflectance spectra (UV-vis DRS) were obtained using an Agilent Technologies Cary 5000 spectrophotometer with an integrating sphere in which BaSO_4_ powder was used as a reference. Photoluminescence (PL) and time-resolved transient PL decay spectra were recorded on Hitachi FL-4600 and Edinburgh FLS1000 fluorescence spectrophotometer with an excitation wavelength of 365 nm, respectively.

### Photocatalytic experimental

The RhB photodegradation was examined as a model reaction to evaluate the photocatalytic performance of the samples. Fifty milligrams of photocatalyst was dispersed in 50 mL RhB solution (5 mg/L) into the quartz photo-reactor vessel. A 500 W Xenon-arc lamp that placed 15 cm away from the reactor was served as the light source. Initially, the mixture was kept in the dark for 30 min under the magnetic stirring to reach the adsorption-desorption equilibrium. Later, aliquots of suspension (4 mL) was sampled and centrifuged at the given intervals time of 30 min. The concentration of dye was analyzed by an Agilent Technologies Cary 5000 spectrophotometer.

For comparison, a certain amount of Bi_4_NbO_8_Cl and ZnTiO_3_ (mass ratio of ZnTiO_3_:Bi_4_NbO_8_Cl = 20%) were added directly into a quartz photo-reactor vessel to do photocatalytic activity evaluation experiment. The result of this sample was named as 20% BNZ-C (“C” means comparison).

The process of capture agent experiment is the same as that of photocatalytic activity evaluation just added respectively 40 μL isopropanol (IPA) as a hydroxyl radical scavenger, 0.005 g p-benzoquinone (BQ) as a superoxide radical scavenger, 0.0158 g ethylenediaminetetraacetic acid disodium salt (EDTA-2Na) as a hole trapping agent, and 0.078 g potassium bromate (KBrO_3_) as an electron trapping agent.

### Electrochemical measurements

The photoelectrochemical properties were measured on a CHI760E electrochemical system (Shanghai Chenhua, China) in a standard three electrode with the catalyst-deposited FTO glass, Pt plate, and Ag/AgCl electrode as the photoanode, counter electrode, and reference electrode, respectively. Meanwhile, 0.5 M Na_2_SO_4_ was used as the electrolyte solution. Transient photocurrent measurements were carried out using a 500 W Xe lamp as a light source. The Mott-Schottky measurement was performed at frequency of 1000 Hz. The working photoanodes were prepared as follows: 30-mg sample, 300 μL mixture solution of chitosan (1% wt%), and acetic acid (1% wt%) were mixed by stirring for 20 min to make a suspension. Then, the mixture above was added dropwise onto an FTO glass (3 × 1 cm) and dried at 40 °C.

## Results and discussion

**Fig. 1:**
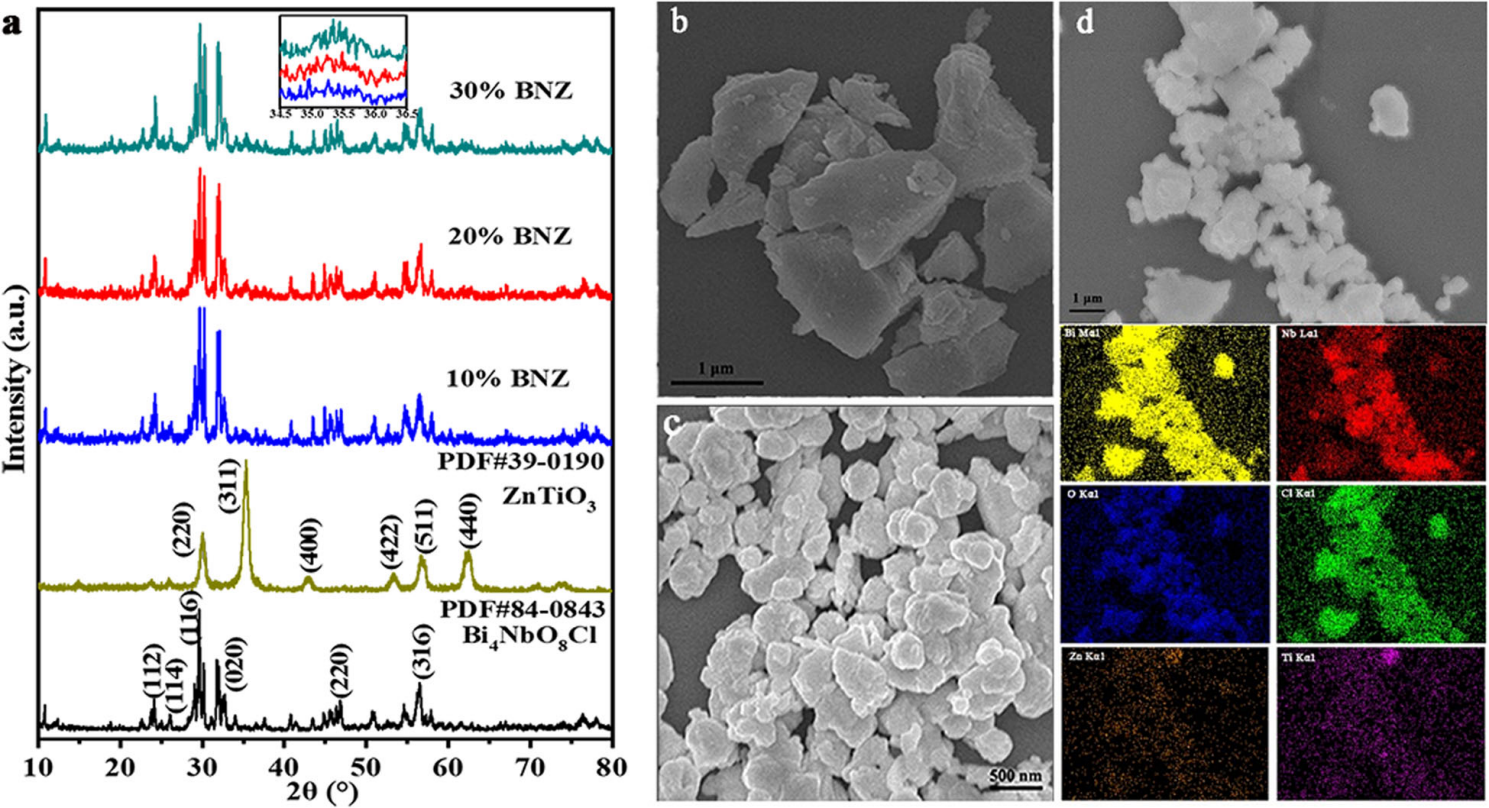
**a** XRD patterns of ZnTiO_3_, Bi_4_NbO_8_Cl, and BNZ samples; SEM images of **b** Bi_4_NbO_8_Cl, **c** ZnTiO_3_, **d** SEM image, and EDX mapping images of 20% BNZ

The crystal structure of the samples could be detected from XRD results [20], as shown in Fig. 1a. The characteristic diffraction peaks at 23.7°, 26.0°, 29.6°, 32.6°, 46.7°, and 56.3°could be indexed to the (112), (114), (116), (020), (220), and (316) planes of the bare Bi_4_NbO_8_Cl (JCPDS card 84-0843). The crystal planes (220), (311), (400), (422), (511), and (440) have a good correspondence with the cubic perovskite ZnTiO_3_ structure (space group R-3 with cell constant a = b = c = 0.841 nm, JCPDS card 39-0190). The XRD patterns of BNZ samples are similar to that of the Bi_4_NbO_8_Cl, and the intensity of the reflection diffraction peak at 35.4° for ZnTiO_3_ increased with the addition of ZnTiO_3_ content. Moreover, the signals associated with Zn, Ti, Bi, Nb, O, and Cl are observed from the EDX mapping images (Fig. 1d) of ZnTiO_3_/Bi_4_NbO_8_Cl heterojunction.

The morphology of ZnTiO_3_, Bi_4_NbO_8_Cl, and BNZ samples are investigated by SEM. Figure 1b shows that ZnTiO_3_ sample is micron-scale irregular blocks structure. Pristine Bi_4_NbO_8_Cl products are composed with irregular ellipsoid particles in which shows stacked structure due to the particles clump together, seen from Fig. 1c. As for 20% BNZ compound (Fig. 1d), it can be found that ZnTiO_3_ are crushed and attached on the surface of the Bi_4_NbO_8_Cl after grinding, ultrasonic mixing, and calcination treatment.

**Fig. 2:**
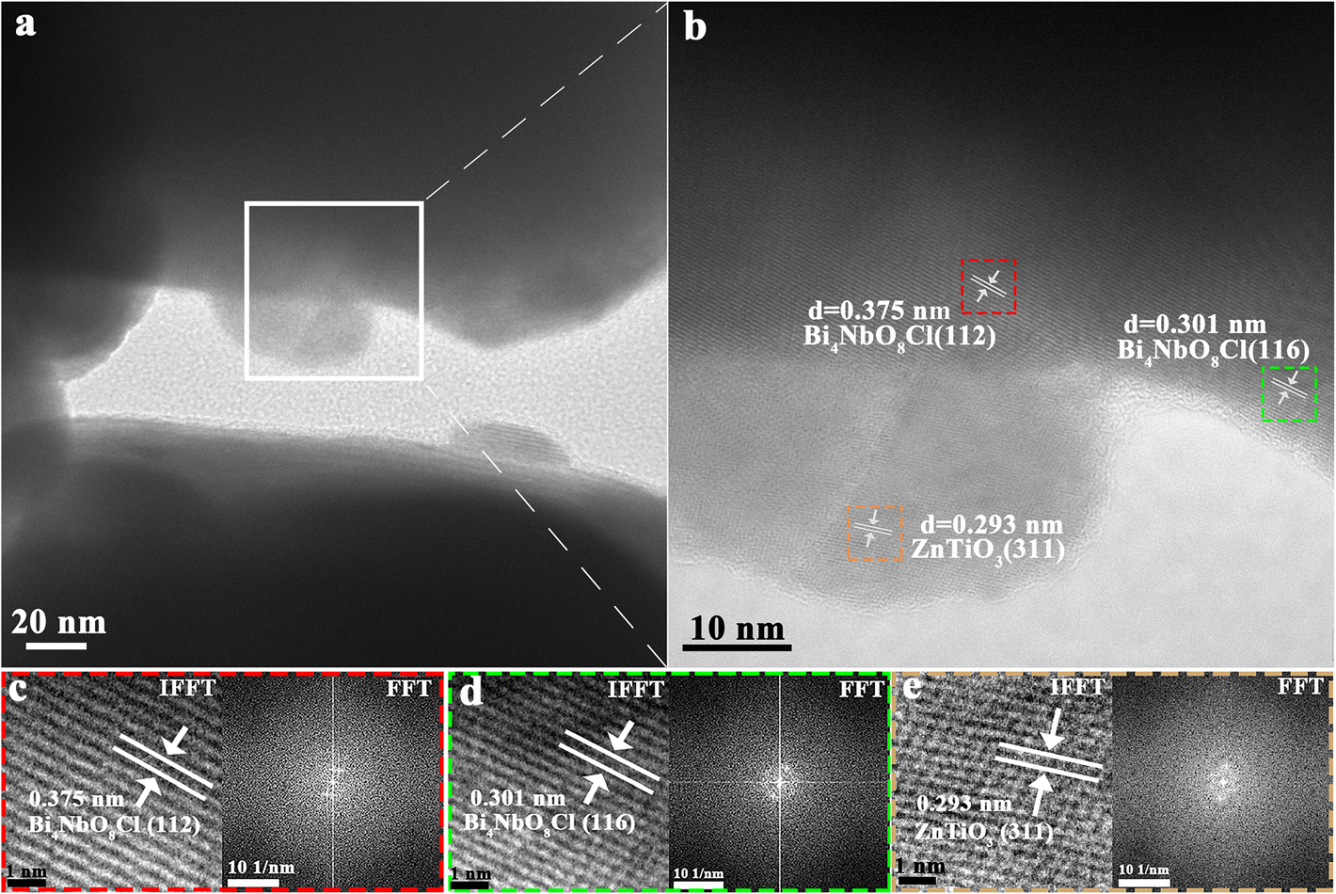
**a**, **b** TEM and HRTEM images of 20% BNZ; **c** IFFT and FFT images of Bi_4_NbO_8_Cl (112) crystal planes; **d** IFFT and FFT images of Bi_4_NbO_8_Cl (116) crystal planes; **e** IFFT and FFT images of ZnTiO_3_ (311) crystal planes

Figure 2 displays the TEM and HRTEM images of the 20% BNZ sample, and the fast Fourier transform (FFT) pattern and inverse FFT (IFFT) image of corresponding select areas. It can be clearly observed that there is a close interface contact between the ZnTiO_3_ blocks and Bi_4_NbO_8_Cl blocks (Fig. 2a). Marked with red wireframe in Fig. 2b, the measured lattice fringe of 0.375 nm is corresponded to Bi_4_NbO_8_Cl (112) crystal plane, and its corresponding FFT and IFFT is displayed in Fig. 2c. As shown in Fig. 2b, the measured lattice fringes of 0.301 nm and 0.293 nm are matched well with Bi_4_NbO_8_Cl (116) crystal plane (green areas) and ZnTiO_3_ (311) crystal plane (orange areas), and their FFT and IFFT images are shown in Fig. 2d and Fig. 2e, respectively. The HRTEM analysis suggests that Bi_4_NbO_8_Cl and ZnTiO_3_ are well combined.

**Fig. 3:**
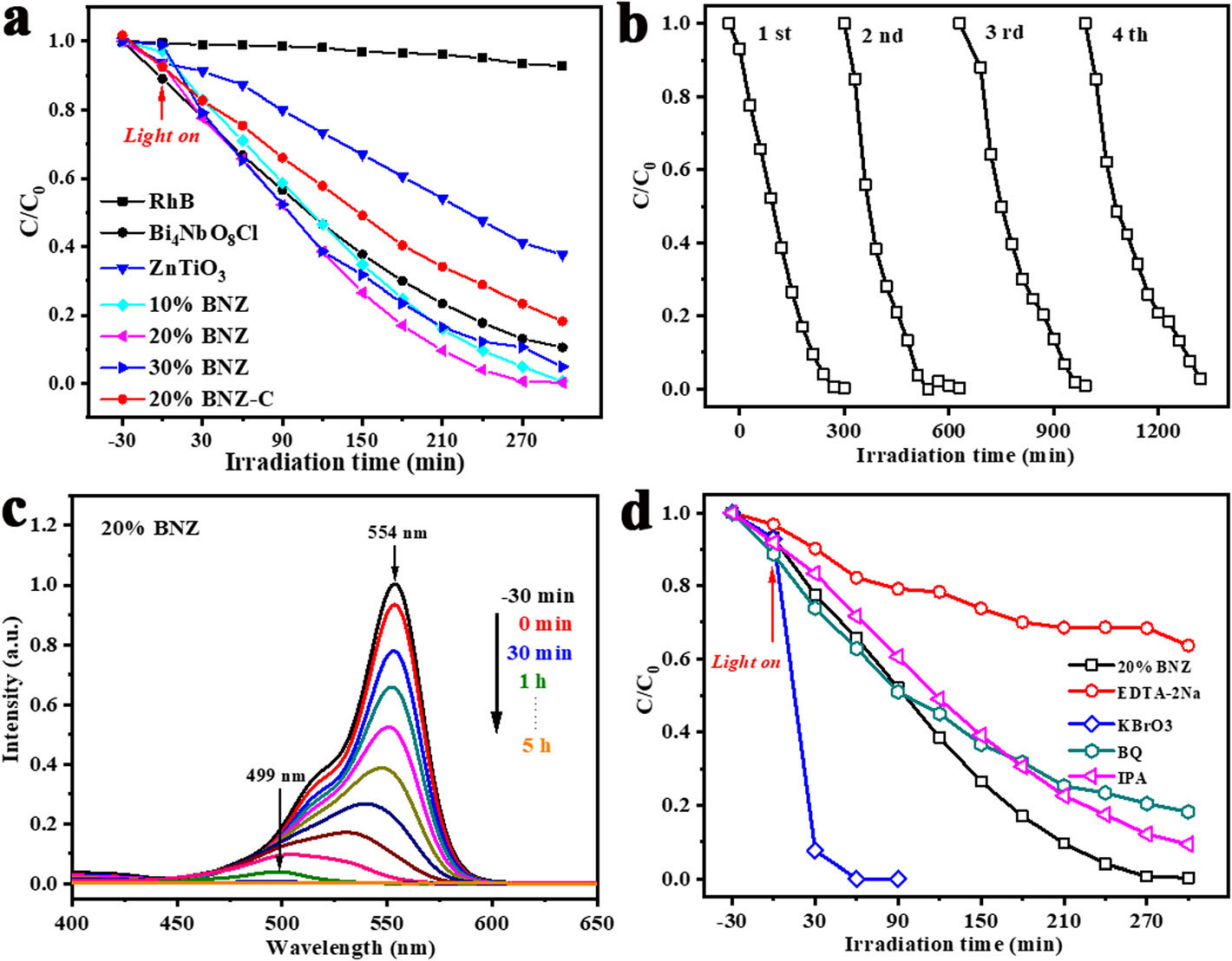
**a** Photocatalytic degradation efficiencies of RhB with ZnTiO_3_, Bi_4_NbO_8_Cl, and BNZ heterojunctions under Xenon-arc lamp irradiation; **b** cycled runs of 20% BNZ; **c** changes in UV-vis absorption spectra of RhB by 20% BNZ under Xenon-arc lamp irradiation; **d** trapping experiments results of 20% BNZ with Xenon-arc lamp irradiation

The photocatalytic performance of pristine Bi_4_NbO_8_Cl, ZnTiO_3_, and BNZ heterojunctions were evaluated by the degradation of RhB dye aqueous solution under Xenon-arc lamp irradiation. As shown in Fig. 3a, the adsorption rate of RhB for all samples are 0–11% in dark. After exposure to light for 5 h, the degradation rate over the bare Bi_4_NbO_8_Cl and ZnTiO_3_ are 89% and 61%, respectively. Moreover, the BNZ composites display improved photocatalytic activity, and the dye removal efficiency is increased with the increase of ZnTiO_3_ contents at first, and then the photodegradation performance has a slight decrease when the ZnTiO_3_ content increased from 20 wt% to 30 wt%. The 20% BNZ composite displays the highest photocatalytic activity with a degradation rate of almost 100%. As for the 20% BNZ-C, the RhB removal rate over it is 81% after 5 h reaction. Twenty percent BNZ showed higher photocatalytic performance due to efficient separation of carriers after heterojunction formation.

The recyclability of the photocatalysts is also an important aspect in their practical application. The cyclic experiments of removing RhB dye were carried out under the same conditions to investigate the recyclability of 20% BNZ samples, as shown in Fig. 3b. After four repeated experiments, the photocatalytic activity just presents a slight decrease, indicating that the 20% BNZ is a stable photocatalyst for the degradation of RhB. Figure 3c shows the changes in UV-vis absorption spectra of RhB by 20% BNZ, with the irradiation time increased, the intensity of characteristic peak is decreased. In addition, the position of absorption peak shifted from 554 to 499 nm during photocatalytic reaction. This blue shift of absorption maximum is caused by the *N*-deethylation of RhB [21–23].

To clarify the main active species responsible for RhB degradation by 20% BNZ composite, the trapping experiments were carried out. The ethylenediaminetetraacetic acid disodium salt (EDTA-2Na), potassium bromate (KBrO_3_), benzoquinone (BQ), and isopropanol (IPA) act as the scavengers of hole (h^+^), electron (e^−^), superoxide radical (•O_2_^−^), and hydroxyl radical (•OH), respectively. As shown in Fig. 3d, the photodegradation rate is affected seriously and decreased by the addition EDTA into the photocatalytic reaction system, and the photocatalytic activity is inhibited slightly when BQ or IPA is added. Hence, the h^+^ was the main dominant reactive species, and the •O_2_^−^ or •OH participated in the degradation process of RhB in 20% BNZ system.

**Fig. 4:**
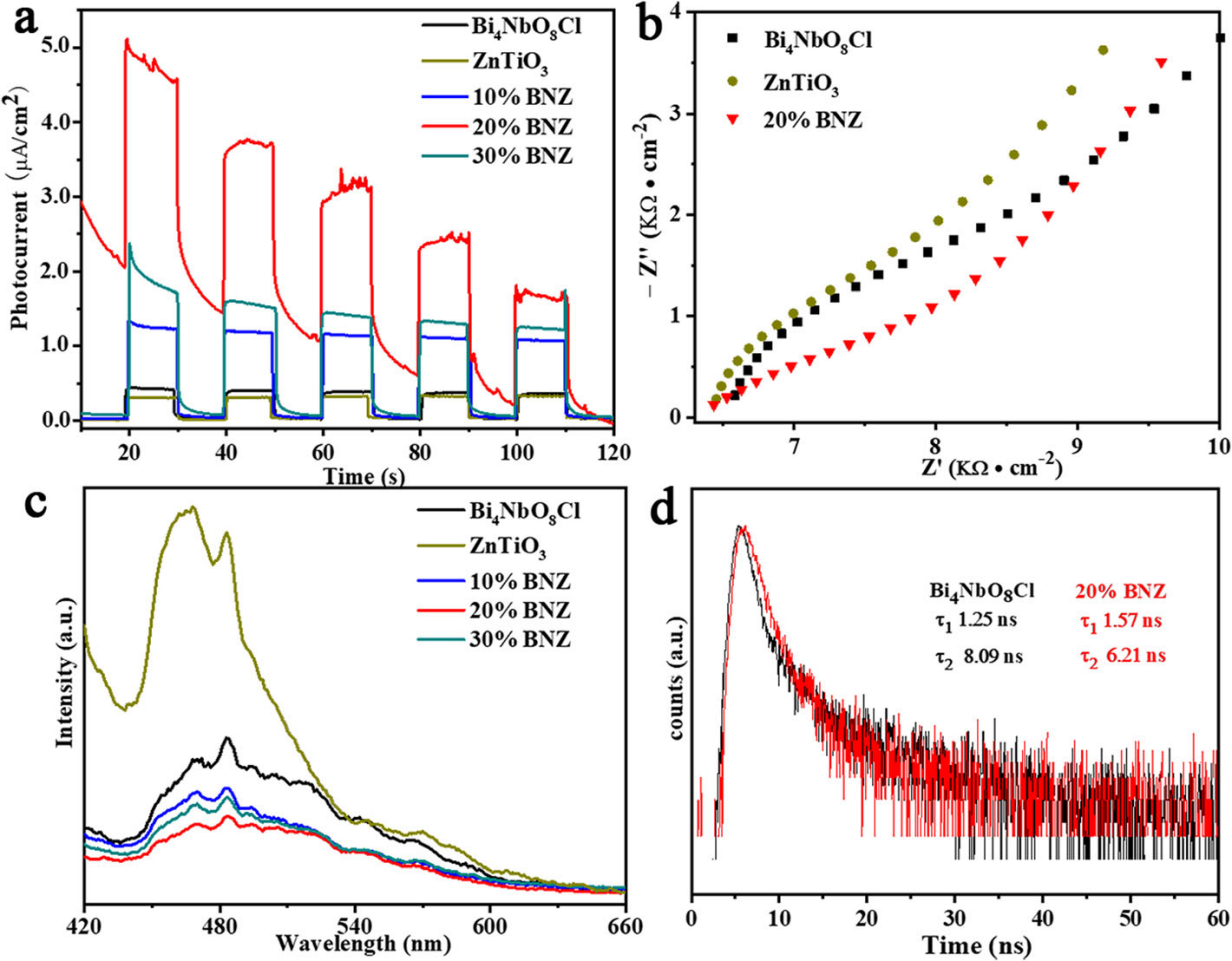
**a** Transient-photocurrent responses of ZnTiO_3_, Bi_4_NbO_8_Cl, and BNZ samples. **b** Electrochemical impedance spectroscopy of ZnTiO_3_, Bi_4_NbO_8_Cl, and 20% BNZ. **c** Photoluminescence spectra of ZnTiO_3_, Bi_4_NbO_8_Cl, and BNZ samples. **d** Time-resolved PL spectra of Bi_4_NbO_8_Cl and 20% BNZ

As shown in Fig. 4a, transient-photocurrent responses of as-prepared photocatalysts were measured under the light irradiation with intermittent on-off cycles to evaluate the production and migration of photogenerated carriers. The higher intensity of photocurrent, the stronger generating ability of photogenerated carriers [24, 25]. The photocurrent density is higher in the light than that in the dark, and displays a typical on-off cycle mode. The photocurrent response intensities obey the follow order: 20% BNZ > 30% BNZ > 10% BNZ > Bi_4_NbO_8_Cl > ZnTiO_3_. It means that the photogenerated carriers’ production ability of 20% BNZ is the best. The obviously enhanced photocurrent density of 20% BNZ sample could be attributed to the intimated contact in the heterojunction, which benefits to the charge generation, separation, and transfer. Moreover, the electrochemical impedance spectroscopy (EIS) was employed to study the ability of the interfacial charge transfer of catalysts. The smaller arc radius of EIS Nyquist plots becomes, the smaller charge transfer resistance is [26]. From the results (Fig. 4b), it can be observed that the 20% BNZ exhibited the smallest semi-circular arc, which indicated that 20% BNZ possesses smaller transfer resistance, and the charge carriers process is very fast in comparison with others as-prepared samples. In order to investigate the recombination behaviors of photogenerated carriers, the PL spectra (Fig. 4c) with the excited wavelength of 365 nm at room temperature were obtained [27]. Compared to bare Bi_4_NbO_8_Cl, the PL intensity of the as-prepared 20% BNZ is weaker, indicating a lower recombination rate of photogenerated carriers. These results imply that the introduction of ZnTiO_3_ could effectively inhabit the recombination of photo-generated electrons (e^−^) and holes (h^+^). As shown in Fig. 4d, time-resolved PL spectra could provide the information about lifetime of photo-excited carriers. The time-resolved PL decay curves of samples were fitted by Eq. (1):

1 $$ I(t)={A}_1{e}^{\frac{-t}{\tau_1}}+{A}_2{e}^{\frac{-t}{\tau_2}} $$

Where *τ*_1_ and *τ*_2_ are the fast decay constant (shorter lifetime) and slower decay constant (long lifetime), respectively. *A*_1_ and *A*_2_ are the corresponding amplitudes. The average lifetime was calculated through Eq. (2) [17]:

2 $$ \uptau =\frac{A_1{\tau}_1^2+{A}_2{\tau}_2^2}{A_1{\tau}_1+{A}_2{\tau}_2} $$

The average lifetime of 20% BNZ is shorter than that of Bi_4_NbO_8_Cl (τ_BiNb_ = 3.66 ns and τ_20%BNZ_ = 2.72 ns). The *τ* value is decreased from 3.66 to 2.72 after modifying ZnTiO_3_, indicating the formation of heterojunction could improve the transfer efficiency of carriers and promote the separation of photogenerated electrons and holes [28–30].

**Fig. 5:**
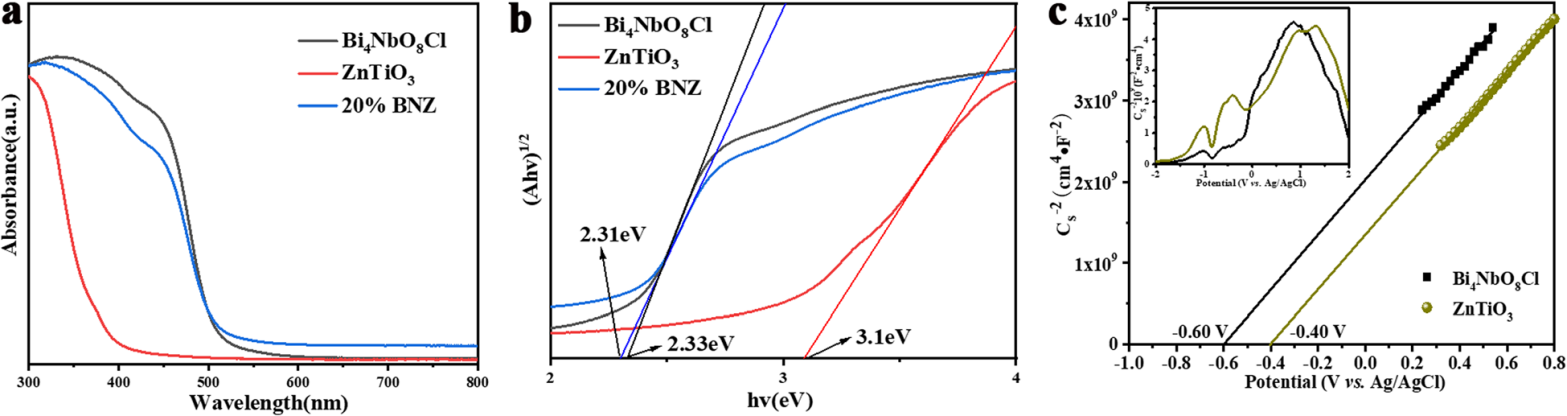
**a** DRS spectra of ZnTiO_3_, Bi_4_NbO_8_Cl, and 20% BNZ sample. **b** Tacus’s curves of ZnTiO_3_, Bi_4_NbO_8_Cl, and 20% BNZ sample. **c** Mott–Schottky curves of ZnTiO_3_ and Bi_4_NbO_8_Cl

Diffuse reflectance spectra (DRS) of Bi_4_NbO_8_Cl, ZnTiO_3_, and 20% BNZ were measured in the range of 300–800 nm to study their optical property. As shown in Fig. 5a, it can be found that the absorption edge of ZnTiO_3_ is 375 nm, and Bi_4_NbO_8_Cl has an intense absorption band with a steep absorption edge at about 505 nm. In addition, the absorption edge 20% BNZ is about 510 nm. Besides, the band gap energy (Eg) of the semiconductors can be calculated by Tauc’s equation, (αhv)^n^ *= A*(hv − Eg), where Eg, *A*, *α*, *h*, and *v* are the band gap, absorption constant, absorption coefficient, Planck’s constant, and light frequency, respectively [31]. In addition, *n* represents a direct-transition material (*n* = 2) or an indirect-transition material (*n* = 1/2). As we all know, both Bi_4_NbO_8_Cl and ZnTiO_3_ are indirect-transition semiconductor, thus *n* equals to 4. As shown in Fig. 5b, the band gap values of as-prepared Bi_4_NbO_8_Cl, ZnTiO_3_, and 20% BNZ samples are 2.33 eV, 3.10 eV, and 2.31 eV, respectively.

Conduction band (CB) potential and valance band (VB) potential are the upmost important factors to understand the heterojunction formation and electron transfer mechanism of nanocomposites. It is known that the bottom of the CB is close to the flat band position; thus, Mott–Schottky tests were carried out to estimate the flat band potential (E_fb_) of samples [32]. Corresponding flat band potential of the electrode were obtained from the M-S plots employing the following Eqs. (3) and (4) [31, 33]:

3 $$ \mathrm{For}\ \mathrm{an}\ \mathrm{n}-\mathrm{type}\ \mathrm{semiconductor}\frac{1}{C^2}=\frac{2}{e\varepsilon {\varepsilon}_o ND}\left(E-{E}_{fb}-\frac{KT}{e}\right) $$

4 $$ \mathrm{For}\ \mathrm{an}\ \mathrm{p}-\mathrm{type}\ \mathrm{semiconductor}\frac{1}{C^2}=\frac{2}{e\varepsilon {\varepsilon}_0 NA}\left(E-{E}_{fb}-\frac{KT}{e}\right) $$

where the *ε*, *ε*_o_, *e*, *C*, *E*, *E*_fb_, *K*, *T*, *N*_D_, and *N*_A_ represent the dielectric constant of materials, permittivity of free space, charge of electron (1.60 × 10^−19^ C), capacitance of the space charge region, applied to the potential, flat band potential, Boltzmann constant, absolute temperature, donor, and acceptor density, respectively. As shown in Fig. 5c, all the plots display positive slops, which confirmed clearly that as-prepared samples act as n-type semiconductor behavior [34, 35]. The flat band potential can be measured from the intersection of linear potential curve up to the *X*-axis at point 1/C^2^ = 0, and can convert to normalized hydrogen electrode scale (NHE) according the formula (5) [36]:

5 $$ E\left(\mathrm{NHE}\right)=E\left(\mathrm{Ag}/\mathrm{AgCI}\right)+0.197\mathrm{V} $$

According to M-S results, the flat band potential for Bi_4_NbO_8_Cl and ZnTiO_3_ is − 0.60 eV and − 0.40 eV (vs. Ag/AgCl), respectively. Accordingly, E_CB_ of Bi_4_NbO_8_Cl and ZnTiO_3_ is − 0.403 eV and − 0.203 eV, respectively. Thus, the E_VB_ of Bi_4_NbO_8_Cl is 1.927 eV, and E_VB_ of ZnTiO_3_ is 2.897 eV.

The specific BET surface areas of the Bi_4_NbO_8_Cl, ZnTiO_3_, and 20% BNZ are shown in Table 1. The S_BET_ of 20% BNZ is 0.87 m^2^/g more than S_BET_ of Bi_4_NbO_8_Cl. It can be seen from Table 1 that the S_BET_ of ZnTiO_3_ is 5.34 m^2^/g. The increased S_BET_ of 20% BNZ is due to the introduction of ZnTiO_3_. The ability to use light of ZnTiO_3_ is weak owning to its wide band gap. Therefore, the increased S_BET_ may not provide many effective active sites. In contrast, ZnTiO_3_ may cover the active sites of Bi_4_NbO_8_Cl surface or becomes new recombination centers of electrons and holes. Thus, the increased S_BET_ of 20% BNZ may only provide a slight impact for enhanced photocatalytic performance. The improved performance is mainly due to the formation of heterojunction.

**Table 1:** Specific BET surface areas parameters of the Bi_4_NbO_8_Cl, ZnTiO_3_, and 20% BNZ samples

Catalyst code	BET surface area (S_BET_) (m^2^/g)
Bi_4_NbO_8_Cl	1.36
ZnTiO_3_	5.34
20% BNZ	2.23

**Scheme 1:**
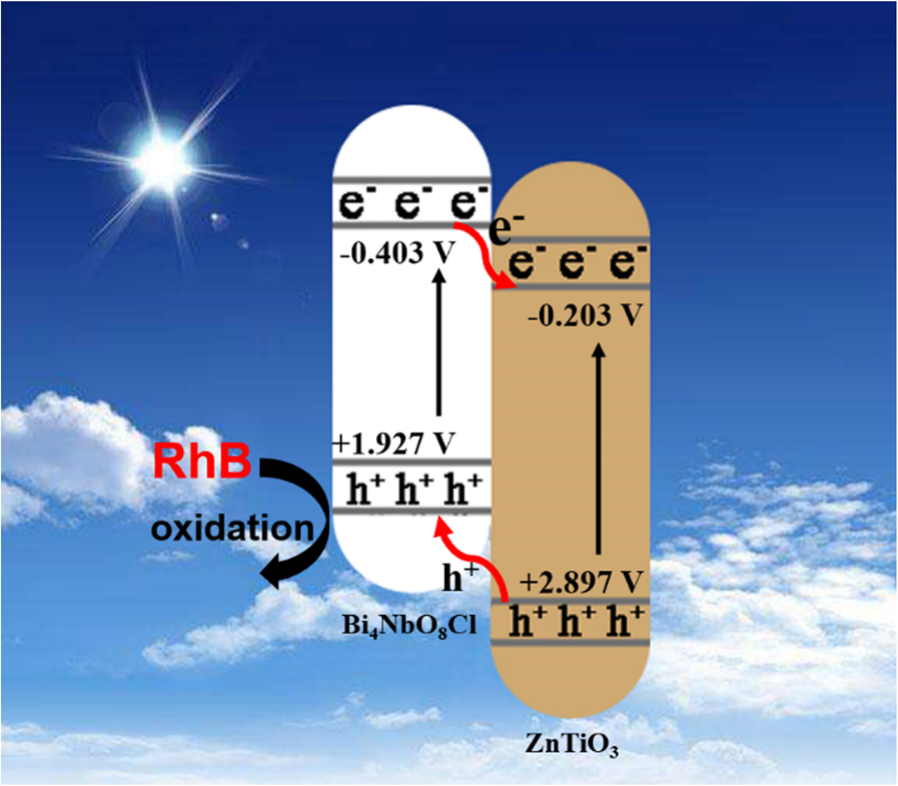
Schematic diagram of the proposed mechanism for the degradation of RhB over the ZnTiO_3_/Bi_4_NbO_8_Cl heterojunction photocatalysts under Xenon-arc lamp irradiation.

To explain the enhanced photocatalytic performance, a possible photocatalytic mechanism is proposed in Scheme 1. Under Xenon-arc lamp irradiation, the electrons (e^−^) are generated in Bi_4_NbO_8_Cl, and they transfer from VB to CB leaving corresponding holes (h^+^) on VB. Meanwhile, the same process takes place in ZnTiO_3_. Through a comparison of energy band potential between Bi_4_NbO_8_Cl and ZnTiO_3_, E_CB_ (Bi_4_NbO_8_Cl) is more negative than E_CB_ (ZnTiO_3_), and E_VB_ (ZnTiO_3_) is more positive than E_VB_ (Bi_4_NbO_8_Cl). Therefore, they can form a type-II heterojunction. Because of the internal electric field, e^−^ on CB of Bi_4_NbO_8_Cl is transferred to CB of ZnTiO_3_, and h^+^ on VB of ZnTiO_3_ is transferred to VB of Bi_4_NbO_8_Cl, realizing the separation of photo-excited e^−^-h^+^ pairs, which leads to the enhancement of performance. Because 20% BNZ has a high positive potential of VB, so its holes have high oxidative capacity. Therefore, holes on VB can directly oxidize organic pollutants like RhB. However, excessive ratio of ZnTiO_3_ in BNZ heterojunction will cover the active sites of Bi_4_NbO_8_Cl surface, decreasing its light-harvesting ability. Moreover, excessive ratio of ZnTiO_3_ may become new recombination centers of electrons and holes. Hence, the quantity of ZnTiO_3_ has an optimum value in heterojunction.

## Conclusions

In this work, the ZnTiO_3_/Bi_4_NbO_8_Cl heterojunction catalyst was prepared successfully via a typical mechanical mixing method. The heterojunction exhibits enhanced photocatalytic performance in comparison with individual ZnTiO_3_ or Bi_4_NbO_8_Cl under Xenon-arc lamp irradiation. Specially, 20% ZnTiO_3_/Bi_4_NbO_8_Cl heterojunction has the best performance. This report may inspire the development of heterojunction structure in catalyst modification and application.

## Data Availability

The datasets used during the current study are available from the corresponding author on reasonable request.

## Abbreviations

BNZ: ZnTiO_3_/Bi_4_NbO_8_Cl
BNZ-C: ZnTiO_3_/Bi_4_NbO_8_Cl-Comparison
S_BET_: Specific BET surface areas
PL: Photoluminescence
CB: Conduction band
VB: Valence band
